# Membrane homeoviscous adaptation in the piezo-hyperthermophilic archaeon *Thermococcus barophilus*

**DOI:** 10.3389/fmicb.2015.01152

**Published:** 2015-10-21

**Authors:** Anaïs Cario, Vincent Grossi, Philippe Schaeffer, Philippe M. Oger

**Affiliations:** ^1^CNRS, Laboratoire de Géologie de Lyon, Ecole Normale Supérieure de Lyon, UMR 5276, Université Claude Bernard Lyon 1Lyon, France; ^2^CNRS, Laboratoire de Biogéochimie Moléculaire, Institut de Chimie de Strasbourg, Ecole de Chimie, Polymères et Matériaux, UMR 7177, Université de StrasbourgStrasbourg, France

**Keywords:** archaeal lipids, archaeal membrane, homeoviscous adaptation, piezophily, deep-biosphere, GDGT-0, archaeol, *Thermococcus barophilus*

## Abstract

The archaeon *Thermococcus barophilus*, one of the most extreme members of hyperthermophilic piezophiles known thus far, is able to grow at temperatures up to 103°C and pressures up to 80 MPa. We analyzed the membrane lipids of *T. barophilus* by high performance liquid chromatography–mass spectrometry as a function of pressure and temperature. In contrast to previous reports, we show that under optimal growth conditions (40 MPa, 85°C) the membrane spanning tetraether lipid GDGT-0 (sometimes called caldarchaeol) is a major membrane lipid of *T. barophilus* together with archaeol. Increasing pressure and decreasing temperature lead to an increase of the proportion of archaeol. Reversely, a higher proportion of GDGT-0 is observed under low pressure and high temperature conditions. Noticeably, pressure and temperature fluctuations also impact the level of unsaturation of apolar lipids having an irregular polyisoprenoid carbon skeleton (unsaturated lycopane derivatives), suggesting a structural role for these neutral lipids in the membrane of *T. barophilus*. Whether these apolar lipids insert in the membrane or not remains to be addressed. However, our results raise questions about the structure of the membrane in this archaeon and other Archaea harboring a mixture of di- and tetraether lipids.

## Introduction

Biological membranes act as barriers to solute diffusion, and play a central role in energy storage and processing via ion gradients. Maintaining optimal membrane function is therefore crucial for the cell. Under physiological conditions, membranes are relatively fluid, disordered liquid-crystalline phases. Perturbations in lipid phase state have profound consequences on membrane structure and function (Lee, 2003, 2004), i.e., clustering of membrane proteins, reduction of membrane protein activity, reduced solute fluxes, or increased permeability to cations and water. Thus, fluctuations of environmental conditions such as variations in temperature, salinity, hydrostatic pressure, or pH pose serious challenges to cells (Hazel, 1995). Based upon the observation that membrane lipids of *Escherichia coli* grown under contrasting temperatures were different (Marr and Ingraham, 1962; Sinensky, 1971) but exhibited almost identical physical properties, Sinensky (1974) established the basis of homeoviscous adaptation. This theory states that membrane lipid composition of an organism varies in order to favor the maintenance of an optimal functionality of the membrane. The main membrane adaptation strategies in bacteria include: (1) the regulation of fatty acid chain length; (2) the regulation of the degree of unsaturation of fatty acids; (3) the regulation of lipid polar headgroups; or (4) the regulation of the proportion of branched fatty acids (Russell and Nichols, 1999). Each of these modifications can shift the phase transition temperature by up to 20°C (Winter, 2002). Thus, by modifying the lipid composition of its membrane, the cell can adapt and maintain its membrane fluidity in a range suitable with its various functions under changing environmental conditions.

Hydrothermal vents discharge high temperature mineral-rich waters (up to 350°C) into the surrounding cold ocean (2–4°C), defining harsh environments characterized by steep physicochemical gradients in pH (3–8), salinity (∼3–7% NaCl) and temperature (2–350°C), in which it is expected that organisms exert a strong resistance to various and multiple stresses. In the deepest part of the oceans, the vent systems are also submitted to high hydrostatic pressures (HHPs) reaching up to 41 MPa at the Ashadze vent site (Zeng et al., 2009). Despite these contrasted environmental conditions, a large diversity of thermophilic and hyperthermophilic prokaryotes has been described, among which hyperthermophilic Archaea from the order *Thermococcales* often dominate (Flores et al., 2011, 2012). Isolates from deep sites demonstrate a clear adaptation to high pressure. Membranes of deep-sea vent organisms are thus expected to be adapted to the physicochemical conditions encountered in these ecosystems, but little is known about how their membrane lipid composition is modified in response to fluctuations of these conditions.

Unlike bacterial lipids, archaeal lipids are composed of phytanyl and/or biphytanyl hydrocarbon chains linked to glycerol by ether bonds, which confer increased rigidity, thermal stability, and reduced permeability to archaeal membranes (Mathai et al., 2001). The diether phospholipid archaeol [2,3-di-*O*-phytanyl-*sn*-glycerol (DPG)] is frequent in Archaea and is the dominant or sole core lipid in some extreme halophilic Archaea where it forms bilayer membranes (Gibson et al., 2005; Xue et al., 2005; Minegishi et al., 2010). Thermophilic and hyperthermophilic Archaea often present bipolar membrane-spanning tetraether lipids which form monolayer instead of bilayer membranes (De Rosa and Gambacorta, 1988). The most common bipolar core lipid is glycerol-dibiphytanyl glycerol tetraether (GDGT-0; sometimes called caldarchaeol). Bipolar lipids pack more tightly than archaeol-based bilayer membranes to form membranes with increased rigidity and low proton leakage at high temperature and acidic pH (Hanford and Peeples, 2002). Homeoviscous adaptation in Archaea has mostly been studied in response to temperature or pH stresses (Oger and Cario, 2013). In strains harboring mixed diphytanyl diether and dibiphytanyl tetraether lipids, the diether-to-tetraether lipid ratio (D/T) has been shown to vary with both stresses (Sprott et al., 1991; Lai et al., 2008; Matsuno et al., 2009; Boyd et al., 2011). For instance, in *Thermococcus kodakarensis*, this ratio decreases from 64% at 60°C to 34% at 93°C (Matsuno et al., 2009). A second mechanism in the homeoviscous adaptation of archaeal membranes involves the regulation of the number of cyclopentane rings in the biphytane hydrocarbon chains of GDGT-0 as a response to pH or temperature variations (De Rosa et al., 1980a; Sugai et al., 2000; Boyd et al., 2011; Oger and Cario, 2013), which increases the packing efficiency of membrane lipids (Gliozzi et al., 1983; Gabriel and Chong, 2000), and consequently increases membrane stability and lowers permeability (Chong et al., 2012). Archaea may also increase the proportion of unsaturated lipids to adapt to temperature variations as seen in *Methanococcoides burtonii*, which accumulates unsaturated archaeol derivatives in response to a lowering in temperature (Nichols et al., 2004). Finally, a fourth adaptive route was observed in *Methanocaldococcus jannaschii*, a methanogen isolated from a deep-sea vent, in which an increase in temperature induces the accumulation of a cross-linked archaeol derivative, called macrocyclic archaeol (Sprott et al., 1991; Kaneshiro and Clark, 1995), along with an increase in GDGT-0. However, the report of the diether lipid archaeol as sole core lipid in the deep-sea hyperthermophiles such as *Methanopyrus kandleri* (Hafenbradl et al., 1996) or *Thermococcus barophilus* (Marteinsson et al., 1999) suggests that alternative homeoviscous mechanisms may exist in Archaea and may play a significant role in the stability and the functionality of archaeal membranes under thermal, pH, or high pressure stresses.

To investigate this point, we have characterized the membrane lipid composition of the model piezo-hyperthermophilic archaeon *T. barophilus* grown under controlled temperature and pressure. We demonstrate that GDGT-0 is a major core lipid in *T. barophilus*, in contrast to previous reports (cf. Marteinsson et al., 1999). We further show that homeoviscous adaptation of the membrane in *T. barophilus* involves the regulation of the D/T ratio, as previously reported for other *Thermococcales*, but also the regulation of the unsaturation level of apolar lipids (unsaturated lycopane derivatives).

## Materials and Methods

### Microorganism and Growth Conditions

*Thermococcus barophilus* strain MP has been isolated from the Snake Pit hydrothermal vent located on the Mid-Atlantic Ridge at a water depth of 3550 m (Marteinsson et al., 1999). Strain MP displays optimal pressure (P), temperature (T), and salinity at 40 MPa, 85°C and 3% of NaCl, respectively. Cells were grown under strict anaerobiosis in 200 ml of Thermococcales rich medium (TRM; Zeng et al., 2009) supplemented with 1% of polysulfide (from a 0.05 mM stock solution; Ikeda et al., 1972). Cultures grown under HHP were carried out in polyethylene bags whereas cultures grown at atmospheric pressure were performed in 500 ml flasks. The effect of P or T on the lipid composition of *T. barophilus* strain MP was evaluated. The cultures were grown under five different conditions: (i) optimal P and T (40 MPa/85°C; OPT); (ii) sub-optimal P (0.1 MPa/85°C; LP); (iii) sub-optimal T (40 MPa/75°C; LT); (iv) supra-optimal P (70 MP/85°C; HP) and supra-optimal T (40 MPa/90°C; HT). The experiments were run in triplicate. To increase experimental reproducibility, all cultures were inoculated with 0.5% (v/v) of a pre-culture grown under optimal conditions to a final concentration of 1.10^6^ cells ml^-1^. Cell biomass and growth phase were monitored by direct cell counts in a Thoma Chamber (depth, 0.01 mm) using a light microscope (BX41, Olympus, France).

### Lipid Extraction

**FIGURE 1:**
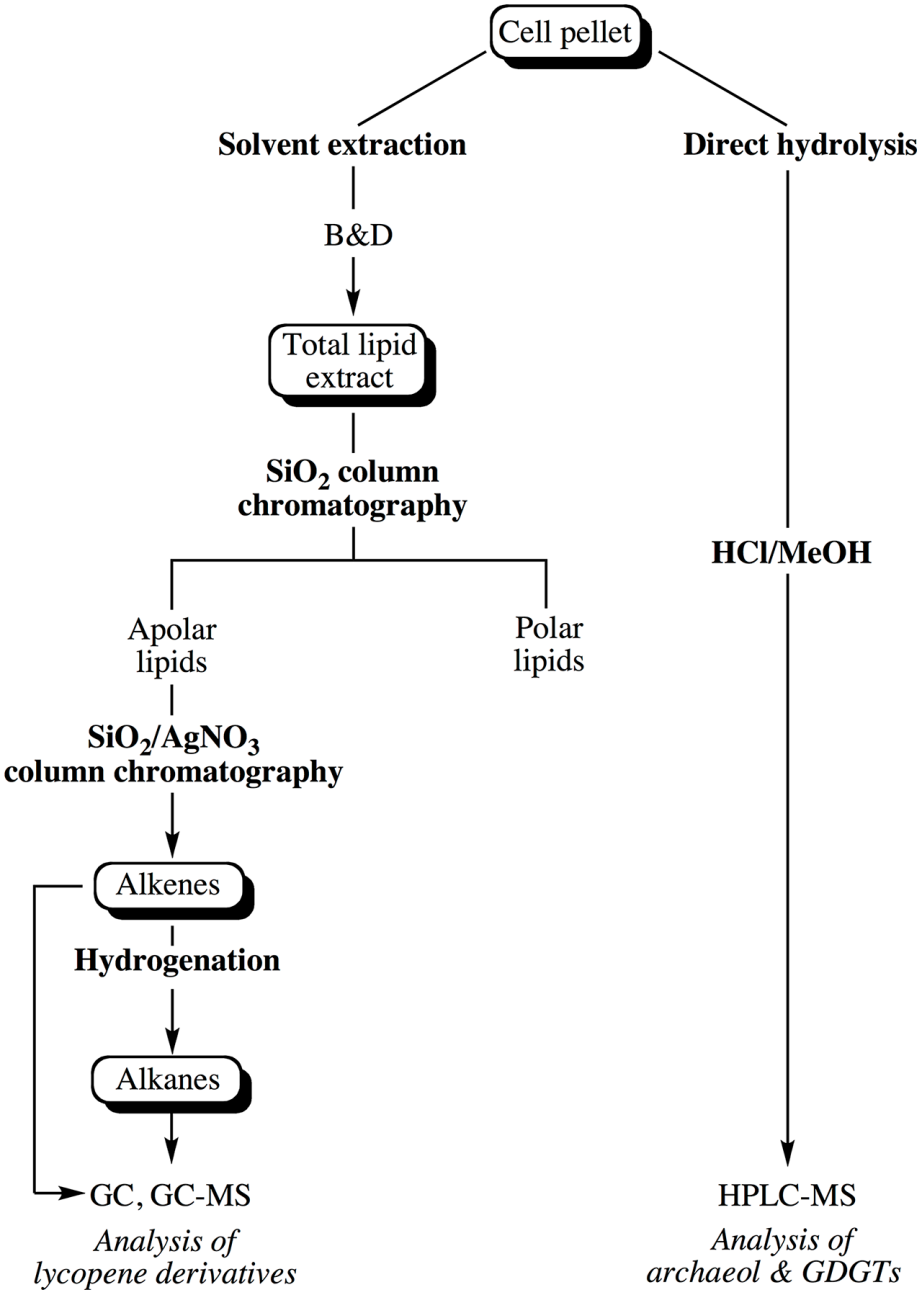
**Experimental scheme showing the different steps considered for the extraction of the apolar and core lipids of *Thermococcus barophilus* grown under different conditions of P and T**.

Lipids were extracted from pellets of cells in late exponential phase harvested by centrifugation (10000 × *g*, 4°C for 20 min) and washed once with an isotonic solution (3% NaCl). The cell pellet was subsequently split into two equal parts in order to extract core lipids and apolar lipids from comparable numbers of cells. The general scheme depicting the extraction and purification of the different classes of lipids is shown in **Figure 1**.

#### Core Lipid Extraction

Core lipids of *T. barophilus* were prepared by direct acid-hydrolysis of half of the cell pellet. Cells were placed in a Pyrex tube, resuspended in a 10% HCl-Methanol (MeOH) solution, and baked for 2 h at 110°C. After cooling, the pH of the solution was adjusted to 5.0 with 2N KOH/MeOH (1:1, v/v). Hydrolyzed lipids were then extracted thrice with *n*-hexane/dichloromethane (DCM; 4:1, v/v), dried over Na_2_SO_4_, evaporated to dryness and resuspended in *n*-hexane/propan-2-ol (99:1, v/v) before being analyzed by high performance liquid chromatography–mass spectrometry following a procedure modified after Hopmans et al. (2000). It is noteworthy that the acid-hydrolysis of cells resulted in the degradation of all the unsaturated C_30_ (squalane), C_35_ and C_40_ (lycopane) isoprenoid derivatives (apolar lipids) for which a separate extraction procedure was considered (see below).

#### Extraction of Total Lipids (TLE) and Separation of Apolar Lipids

Typically, the TLE was obtained from half of the cell pellet using a slightly modified Bligh and Dyer method (B&D; Bligh and Dyer, 1959). Cells were first resuspended in pure water (pre-extracted with DCM), transferred to a glass tube and MeOH and DCM were added to achieve a final ratio of 1:2:0.8 DCM/MeOH/water (v/v/v). The suspension was sonicated 5 min with a probe and centrifuged at 4500 rpm for 10 min. The supernatant was collected and the extraction of the cells was repeated twice. The three respective supernatants were combined and the volume was adjusted to a final DCM/MeOH/water ratio of 1:1:0.9 (v/v/v) allowing the separation of two phases. The lower organic phase was collected and the water phase was further extracted twice with DCM. The three respective organic phases were combined and concentrated by rotary evaporation to yield the TLE. Elemental sulfur (S_0_) present in the TLE was removed with activated copper overnight at 4°C. The TLE was then separated into an apolar and a polar lipid fraction by silica column chromatography using *n*-hexane/DCM (4:1, v/v, apolar lipids) and DCM/MeOH (1:1, v/v, polar lipids), respectively. The apolar fraction was further separated into saturated and unsaturated compounds using a column of silica impregnated with silver nitrate (SiO_2_, AgNO_3_, 20%, w/w). Elution with *n*-hexane and ethyl acetate yielded the saturated hydrocarbons and the total alkene fraction, respectively. Hydrogenation of half of the alkene fraction was performed under a hydrogen atmosphere in ethyl acetate containing a drop of acetic acid and Pd/CaCO_3_ as catalyst.

#### Tests of Lipid Extraction Efficiency in *T. barophilus*

To assay the efficiency of the extraction procedures on the recovery of *T. barophilus* lipids, several commonly used procedures were tested. Three total lipid extractions were performed on independent cell pellets: (1) the classical B&D extraction, (2) the trichloroacetic acid acidified B&D extraction (TCA B&D) proposed by Nishihara and Koga (1987) to increase lipid extraction efficiency from thermoacidophiles, and (3) extraction with DCM and MeOH (1:1, v:v; four times). Following the extraction step, TLE were further purified as described above to remove elemental sulfur. Lipid yields are expressed as percent of the dry weight of cells (cdw). The ratio of di- to tetraether (D/T) was estimated by liquid chromatography-mass spectrometry (LC–MS; see below) after acid hydrolysis of the extracts and compared to the ratio obtained by direct hydrolysis of cell pellets under acidic or basic conditions.

### GC–MS and LC–MS Analyses of Lipid Extracts

Apolar lipids were analyzed by gas chromatography–mass spectrometry (GC–MS) using a MD800 Voyager spectrometer operating at 70 eV and interfaced to an HP6890 gas chromato graph equipped with an on-column injector and a DB-5MS column (30 m × 0.25 mm × 0.25 μm). The oven temperature was programmed from 60°C (1 min) to 130°C at 20°C min^-1^, then to 300°C at 4°C min^-1^ (30 min). Helium was used as the carrier gas at constant flow (1 ml min^-1^). Quantitation of unsaturated hydrocarbons was performed using squalane as external standard.

Hydrolyzed core lipids of *T. barophilus* were analyzed by LC–MS using an HP 1100 series LC–MS instrument equipped with an auto-injector and a Chemstation chromatography manager software. The analytical conditions were modified after Hopmans et al. (2000). Separations were achieved on a Prevail Cyano 3 microns column (150 mm × 2.1 mm; Grace Davison Discovery Sciences) maintained at 30°C. Injection volumes varied from 5 to 10 μl. Archaeol and GDGT-0 were eluted in the same run with a flow rate of 0.8 ml min^-1^, using a linear gradient from 0.1% A and 99.9% B to 1.3% A in 30 min (A = propan-2-ol, B = *n*-hexane), followed by a gradient to 10% A in 5 min (10 min isocratic), and back to 0.1% A in 5 min. Detection was achieved using an Esquire 3000+ ion trap mass spectrometer with an atmospheric pressure positive ion chemical ionization source (APCI-MS). Conditions for MS analyses were: nebulizer pressure 50 psi, APCI temperature 350°C, drying temperature 300°C, drying gas (N_2_) flow five l min^-1^ and temperature 300°C, capillary voltage -2 kV, corona 4 microamperes, scan range *m/z* 600–1400. Mass spectra were systematically background-corrected.

## Results

### Lipid Composition of *T. barophilus* Grown under Optimal Conditions

**FIGURE 2:**
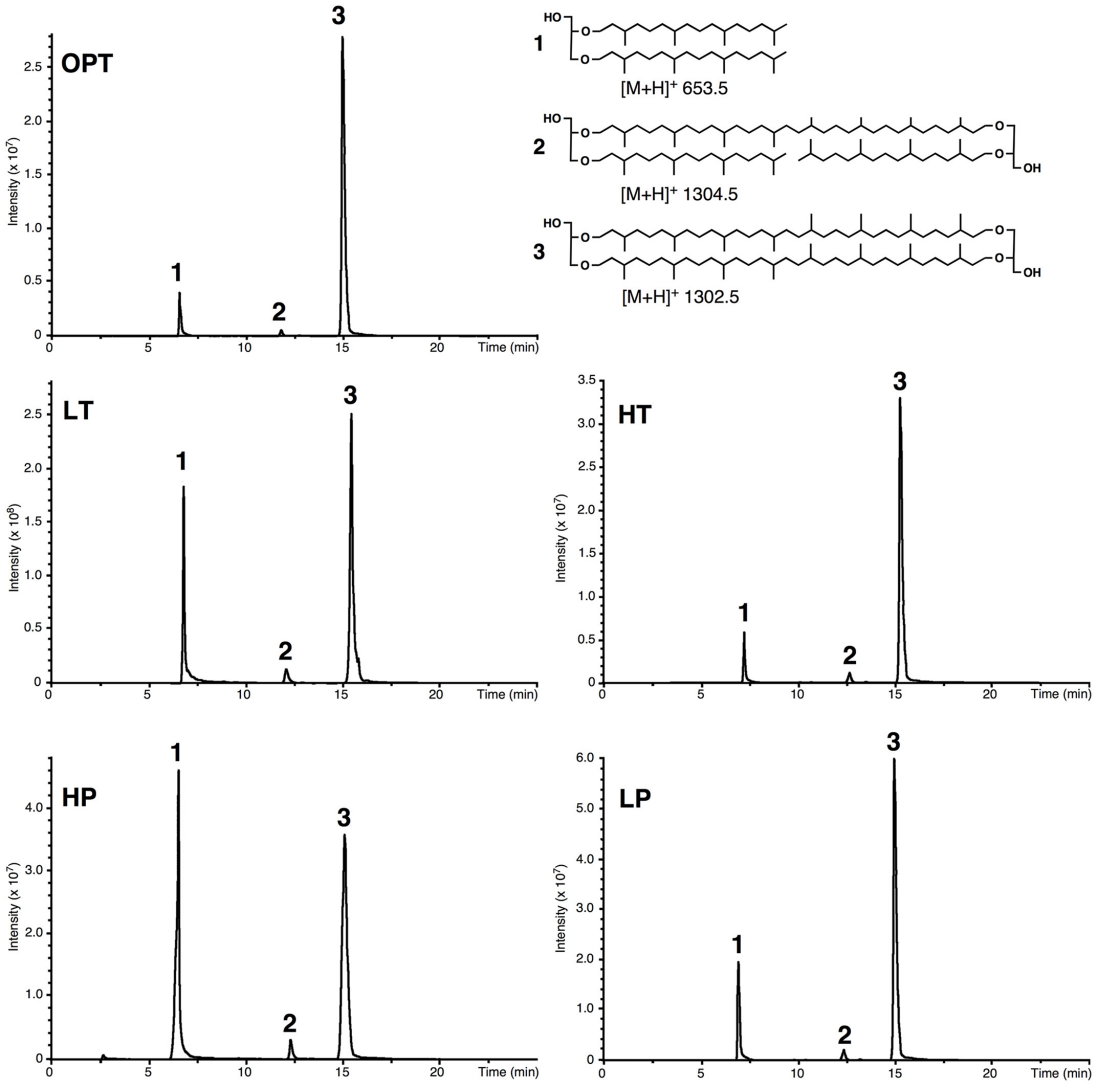
**Example of high performance liquid chromatography–mass spectrometry (HPLC–MS) chromatograms of the lipid extract of acid-hydrolyzed cells of *T. barophilus.*** Cells were grown at optimal pressure and temperature (OPT, 40 MPa, 85°C), low temperature (LT, 40 MPa, 75°C), high pressure (HP, 70 MPa, 85°C), low pressure (LP, 0.1 MPa, 85°C), or high temperature (HT, 40 MPa, 90°C). (1) Diphytanyl glycerol diether (archaeol); (2) glycerol trialkyl glycerol tetraether (GTGT); (3) glycerol dibiphytanyl glycerol tetraether (GDGT-0).

Acid-hydrolyzed core lipids of *T. barophilus* grown under optimal growth conditions (OPT) were analyzed by HPLC–APCI–MS. The major core lipids identified were GDGT-0 ([M+H]^+^ 1302.5; compound 3, **Figure 2**) and archaeol ([M+H]^+^ 653.5; compound 1, **Figure 2**). A third core lipid present in trace amounts showed a protonated [M+H]^+^ ion at *m/z* 1304.5 and was identified as a glycerol-trialkyl-glycerol-tetraether (GTGT; compound 2, **Figure 2**). The latter compound, which contains one biphytanyl and two phytanyl chains, has been formerly proposed to be an intermediate of GDGT-0 biosynthesis (Schouten et al., 2000; Pitcher et al., 2011), although this has been recently subject to debate (Villanueva et al., 2014). H-shaped GDGT-0 derivatives previously reported in several members of the *Thermococcales* (Sugai et al., 2004), GDGT with 1–4 cyclopentane ring(s) observed in some thermoacidophilic Archaea and sulfur-dependent thermophiles (De Rosa et al., 1980b), and macrocyclic archaeol reported in two species of *Methanococcus* (Comita et al., 1984; Trincone et al., 1992), were not detected in *T. barophilus* strain MP. It is noteworthy that the response factors in MS of archaeol and GDGT-0 are not known but that they likely differ due to the higher number of protonable sites present in GDGT-0 compared to archaeol. Thus, the variations of the archaeol/GDGT-0 (D/T) ratio observed between growth conditions (**Figure 2**) indicate qualitative rather than quantitative changes between both classes of core lipids. Nonetheless, considering exclusively the two predominant compounds (i.e., archaeol and GDGT-0) present in the HPLC chromatogram, the area of the peak corresponding to GDGT-0 represented 84% of the ether lipids vs. 16% for archaeol.

**FIGURE 3:**
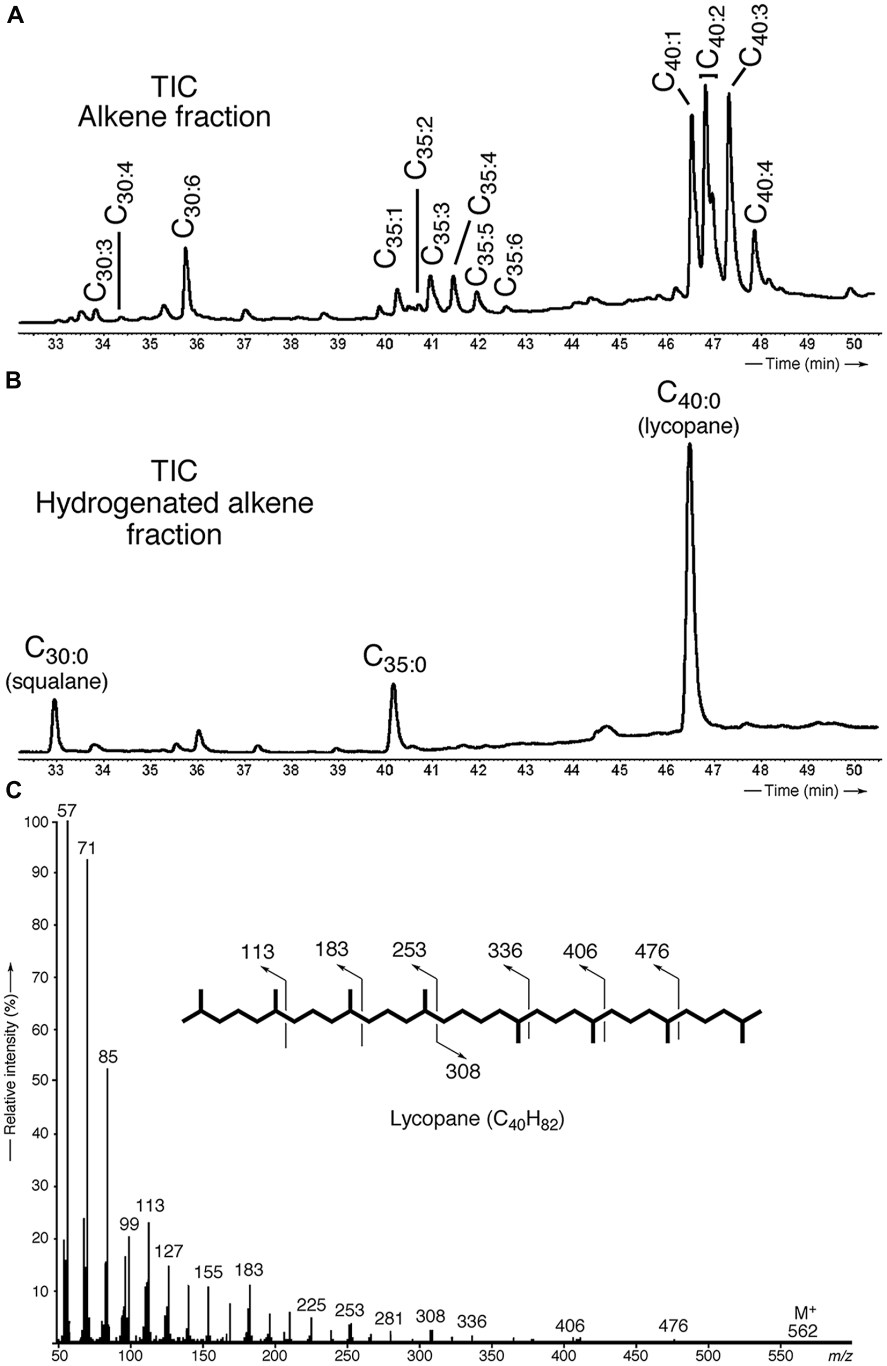
**Identification and characterization of polyunsaturated irregular isoprenoids in *T. barophilus*. (A)** Partial gas chromatogram [total ion current (TIC)] showing the distribution of the polyunsaturated isoprenoids C_30_ (squalane), C_35_ and C_40_ (lycopane) derivatives; **(B)** Partial gas chromatogram (TIC) of a hydrogenated apolar lipid fraction; **(C)** Mass spectrum of lycopane following complete catalytic hydrogenation of the C_40_ lycopene derivatives.

Apolar lipids of *T. barophilus* grown under optimal conditions were detected only in the non-hydrolyzed TLE since they were destroyed upon acid hydrolysis. GC–MS analysis of the alkene fraction showed the presence of a main group of unsaturated hydrocarbons which were identified, based on their mass spectra, as C_40_ isoprenoid alkenes with one up to six double bonds. Two minor groups of C_30_ and C_35_ isoprenoid alkenes with 1–6 double bonds were also detected in trace amounts (**Figure 3A**). Catalytic hydrogenation of the apolar fraction confirmed that each group of alkenes share respectively the same C_40_, C_35_, and C_30_ hydrocarbon skeleton, which could be identified based on their characteristic mass spectral fragmentations as irregular isoprenoids (**Figures 3B,C**), namely lycopane (2,6,10,14,19,23,27,31-octamethyldotriacontane), 2,6,10,14,19,23,27-hepta methyloctacosane and squalane (2,6,10,15,19,23-hexamethyl tetracosane), respectively. C_35_ and C_30_ isoprenoids, present in low abundance in comparison to the C_40_ alkenes, were not considered further in this study. Unsaturated lycopane-derivatives with three and four double bonds were the major isoprenoid hydrocarbons present in *T. barophilus* (**Table 1**; **Figure 3A**). Due to the high number of double bonds in these C_40_ compounds and the low amounts of these compounds per cell, the exact position and the stereochemistry of the double bonds could not be determined. The relative proportion of the lycopene derivatives was estimated to represent ca. 1–2% of total lipids of *T. barophilus* under optimal growth conditions.

**Table 1:** Proportions and average number of double bonds of individual unsaturated lycopane derivatives biosynthesized by *Thermococcus barophil*us grown under different conditions of temperature and pressure.

Culture conditions	Relative proportions of C_40_ lycopane derivatives (%)						Average number of unsaturations
	C_40:1_	C_40:2_	C_40:3_	C_40:4_	C_40:5_	C_40:6_	
HT	28.8	34.5	26.2	10.5	ND	ND	2.2
LP	16.0	39.5	27.4	17.1	ND	ND	2.5
OPT	10.3	21.5	37.4	30.8	Tr	ND	2.9
LT	ND	7.3	27.2	55.1	8.5	1.9	3.8
HP	ND	10.5	28.6	45.9	11.2	3.8	3.7

*ND, not detected; Tr, trace amounts; HT, high temperature (40 MPa, 90°C); LP, low pressure (0.1 MPa, 85°C); OPT, optimal pressure and temperature (40 MPa, 85°C); HP, high pressure (70 MPa, 85°C); LT, low temperature (40 MPa, 80°C)*.

### Influence of Pressure and Temperature on the Lipid Composition of *T. barophilus*

The relative proportions of archaeol and GDGT-0 were determined and compared as a function of growth pressure and temperature, using suboptimal and supraoptimal conditions of pressure (0.1 and 70 MPa) or temperature (75 and 90°C), which have been shown previously to generate the same level of stress in cells of *T. barophilus* (Marteinsson et al., 1999). GTGT (compound 2, **Figure 2**) was observed only in trace amounts under all conditions tested. The D/T ratio significantly varied as a function of growth temperature or pressure (**Table 2**). It increased from 8/92 at HT to 45/55 at LT, which corresponds to a ca. 10-fold increase. The D/T ratio under LP conditions slightly decreased in comparison to optimal conditions from 12/88 to 16/84 respectively, whereas it was much higher under HP conditions reaching 37/63, corresponding to a ca. sevenfold increase in comparison to OPT conditions.

**Table 2:** D/T ratio of core lipids in *T. barophilus* as a function of pressure and temperature.

	D/T
LT	45/55 ± 0.09
HP	37/63 ± 0.14
OPT	16/84 ± 0.04
LP	12/88 ± 0.03
HT	8/92 ± 0.02

*Note that the relative proportions of archaeol and GDGT-0 have been determined by integration of the [M+H]^+^ peaks on the HPLC chromatograms using arbitrarily a 1:1 response factor for both compounds. The values are averages from at least three independent biological replicate experiments*.

**FIGURE 4:**
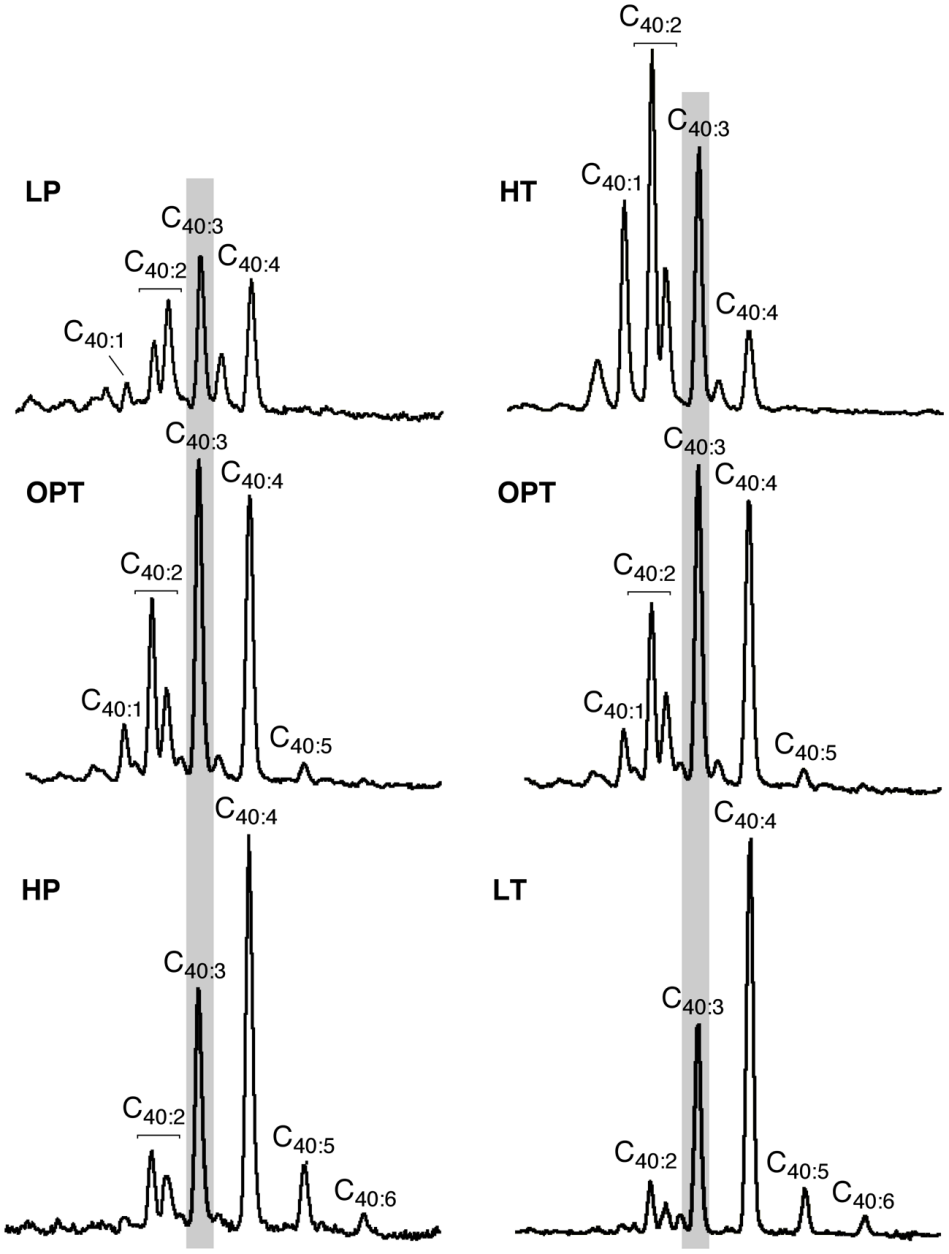
**Variations in the mean unsaturation level of lycopane derivatives in *T. barophilus***. Partial GC–MS chromatograms (TIC) of the isolated apolar lipids of *T. barophilus* as a function of pressure (P) and temperature (T). 40:X indicates a compound with a lycopane C_40_ carbon skeleton and X double bond(s). The major peak observed under optimal growth conditions is highlighted by a light gray background.

In addition to variations in the relative proportions of the two major core lipids, the degree of unsaturation of lycopane derivatives synthesized by *T. barophilus* was also observed to vary according to conditions of pressure or temperature (**Table 1**; **Figure 4**). The average number of double bonds in unsaturated lycopane derivatives increased from 2.9 to 3.7 when the growth pressure was increased from 40 to 70 MPa, and decreased to 2.5 at atmospheric pressure. The effect of temperature mirrored that of pressure, with the average number of unsaturation in lycopane derivatives increasing to 3.8 at low temperature (80°C), and decreasing to 2.2 at high temperature (90°C; **Table 1**; **Figure 4**).

### Polar Lipid Extraction Efficiency in *T. barophilus*

The previously reported absence of tetraether lipids in *T. barophilus* (Marteinsson et al., 1999) prompted us to evaluate the lipid extraction efficiency of different extraction protocols on the archaeal lipids from this species. To address this question, we compared the yield and composition of lipids extracted from *T. barophilus* (cells grown under optimal conditions) using three different procedures of extraction with solvents (B&D, TCA B&D, and DCM/MeOH) and direct acid-hydrolysis (Supplementary Figure S1). *T. barophilus* cells appeared extremely resistant to solvent extraction as evidenced by the poor yields of lipids obtained using the standard or acidified (TCA) B&D procedures (**Table 3** and Supplementary Figure S2). A better yield of TLE, i.e., 3.7% cdw, was obtained with a monophasic extraction using a mixture of DCM/MeOH (2:1, v/v). This yield was still lower than yields reported for *Thermococcales* in the literature, which range between 5 and 10% cdw (Langworthy, 1982). In fact, residual lipids were detected in significant amounts after acid hydrolysis of the residue from B&D pre-extracted cells, confirming incomplete lipid extraction using sonication (results not shown). Direct acid or base hydrolysis of the cell pellets yielded 5.0 and 1.4% of lipids cdw, respectively (**Table 3**). The yield obtained for the acid hydrolysis (5% cdw) is in good agreement with the highest values reported in the literature (10% cdw), considering acid hydrolized lipids lack the polar headgroup moieties and are therefore ca. twice lighter than the intact polar lipids obtained by the B&D procedure. The absence of the polar headgroup results in the formation of relatively low-polar core lipids, the latter being more easily solvent-extractable, and can explain the higher yield obtained in the case of the acid hydrolysis procedure. In addition to yields, lipid extraction procedures also affected core lipid compositions. For example, the B&D and AH procedures yielded almost the same amount of archaeol (data not shown). However, archaeol represented ca. 90% of the total core lipids extracted by the B&D method compared to 16% with the direct hydrolysis of cell pellet method (**Table 3**). Acidification of the extraction solvent using TCA, which has been recommended for the extraction of lipids of hyperthermophilic acidophiles by Nishihara and Koga (1987), neither increased the extraction yield of lipids from *T. barophilus* nor corrected the core lipid composition bias (**Table 3**). The basic hydrolysis of membrane lipids yielded poor results, both in terms of yield (1.4% cdw) and quality, e.g., altered D/T ratio (**Table 3**).

**Table 3:** Extraction yields of total intact lipids and hydrolyzed polar lipids (core lipids) from mid exponential phase cells of *T. barophilus* grown under optimal conditions (3% salinity, 85°C, 40 MPa).

	Solvents used for lipid extraction	Type of lipids extracted	% cdw	Number of replicates	D/T
B&D	BiphasicMeOH/DCM/H_2_O(1:1:0.9, v/v/v)	Intact polar and apolar lipids^∗^	1.2 ± 0.5	*n* = 5	90/10
TCA	BiphasicMeOH/DCM/TCA 5%(1:1:0.9, v/v/v)	Intact polar and apolar lipids^∗^	0.8 ± 0.3	*n* = 3	98/2
DCM/MeOH	MonophasicDCM/MeOH (2:1, v/v)	Intact polar and apolar lipids^∗^	3.7 ± 1.3	*n* = 4	60/40
AH	HCl/MeOH(10% HCl 12N in MeOH)	Core lipids	5.0 ± 0.5	*n* = 5	16/84
BH	1N KOH in MeOH/H_2_O(1:1, v/v)	Core lipids and apolar lipids	1.4 ± 0.2	*n* = 2	6/94

*Yields are given as percent of cell dry weight (cdw)*.

*B&D, Bligh and Dyer; TCA, TCA acidified B&D; DCM+MeOH; dichloromethane:methanol, 1:1, v/v; AH, acid hydrolysis; BH, base hydrolysis; DCM, dichloromethane; MeOH, methanol; D/T, diether/tetraether ratio (archaeol/GDGT-0)*.

*^∗^Half of the lipid extract is further hydrolyzed (HCl/MeOH) prior to LC–MS analysis of ether lipids*.

## Discussion

### GDGT-0 and Archaeol as Major Core Lipids of *T. barophilus*

We investigated the membrane lipid composition and modification in response to variations in P and T in the piezo-hyperthermophilic archaeon *T. barophilus* strain MP, a strain capable of growth up to 103°C and 80 MPa. Our results first establish that the tetraether lipid GDGT-0 is one of the two major core lipids in *T. barophilus*. Thus far, this species has been considered to synthesize only archaeol and not GDGT-0 (Marteinsson et al., 1999). Its abundance relative to archaeol varies according to varying environmental conditions, i.e., temperature and pressure. The high proportion of GDGT-0 among the ether lipids of *T. barophilus* is in good agreement with the ability of this strain to grow at temperatures close to or above 100°C. Indeed, bipolar tetraether lipids, which harbor hydrophilic moieties at both ends and long hydrophobic central domains, are synthesized by all hyperthermophilic and almost all thermophilic Archaea, and have been proposed to constitute an adaptive trait for growth at high temperature. Tetraether-based monolayer membranes exhibit a very high thermal stability and extreme rigidity (Elferink et al., 1994; Dante et al., 1995) and, consequently, a low permeability to water and ions. This is also consistent with the reported core lipid compositions of other *Thermococcales*, which optimal growth temperatures are distributed between 75 and 103°C and which all synthesize significant proportions of tetraether lipids (Sugai et al., 2004). Thus far, the lack of bipolar lipids in *Thermococcales* has been reported only in membranes of *Pyrococcus woesei* (Lanzotti et al., 1989) and *Thermococcus celer* (De Rosa et al., 1987) in addition to *T. barophilus* (Marteinsson et al., 1999), which is rather in contradiction with the stability of the archaeol-based lipid bilayer required for growth at high temperature. Membrane lipid compositions of several Archaea have been shown to vary with the cell growth phase and the growth medium composition (Matsuno et al., 2009; Meador et al., 2014). These studies demonstrated a strong impact of the growth phase on the relative proportions of di- vs. tetraether lipids, but neither study reported the complete absence of one of these two lipid pools, suggesting that the lack of tetraethers in these species may stem from technical issues. Later analyses of *T. celer* and *P. woesei* indeed showed that GDGT-0 is a major lipid of these species (Sugai et al., 2004), which is consistent with the proposed role played by GDGT-0 in membrane stability of hyperthermophiles. Similar discrepancies in whole cell lipid compositions exist in the literature for other *Thermococcales*, such as *T. kodakarensis* (Sugai et al., 2004; Meador et al., 2014; Bauersachs et al., 2015). We hypothesized that these differences may stem from the distinct lipid extraction procedures applied in the different studies, e.g., direct hydrolysis of polar headgroups followed by core lipid extraction (this study and that of Sugai et al., 2004) or intact polar lipid extraction by B&D followed by polar headgroup hydrolysis. To test this hypothesis, we evaluated the ability of different lipid extraction protocols on *T. barophilus* cells (Supplementary Figure S2 and **Table 3**). Our results clearly show that intact polar tetraether lipids are very poorly extracted from *T. barophilus* cells with the B&D procedure, as indicated by the D/T ratio of 90/10 obtained upon hydrolysis of the lipid extract which shows that diethers are preferentially recovered over GDGT-0 (**Table 3**). The exact lipid extraction and lipid analysis procedures used in the original *T. barophilus* description is unknown since not reported by the authors. It is, however, likely that it followed the widely used B&D or TCA approaches. In our hands, the use of the TCA extraction lead to the lowest yields (0.8% cdw) and almost no tetraether were extracted (D/T = 98/2). These poor yields could explain why the original study did not report the presence of polar tetraether lipids in *T. barophilus* (Marteinsson et al., 1999). Consistent with these observations, acid hydrolysis of the residue of cells pre-extracted using the B&D procedure yielded significant amounts of GDGT-0 and of archaeol (data not shown). Noticeably, although the monophasic DCM/MeOH approach yielded the best results, none of the protocols tested appeared able to extract the totality of the polar lipids of *T. barophilus*. Altogether, these observations suggest that results of intact polar lipid compositions should be regarded with caution for certain archaeal species, and evaluated on a strain-to-strain basis using different extraction procedures.

### Evidence for Homeoviscous Adaptation in *T. barophilus*

The modification of the D/T ratio observed when *T. barophilus* was submitted to temperature variations is in good agreement with that previously reported for Archaea harboring mixed diphytanyl diether and dibiphytanyl tetraether lipids, such as *T. kodakarensis* (Sprott et al., 1991; Matsuno et al., 2009; Meador et al., 2014) or *Archaeoglobus fulgidus* (Sprott et al., 1991; Lai et al., 2008). A decrease of the D/T ratio is expected to result in the stabilization of the membranes since it will form monolayers. In monolayer domains, the lipid packing is increased, which helps to regulate the flux of solutes and protons across the membranes. Conversely, an increase of this ratio is expected to increase membrane fluidity and permeability (Chong et al., 2012). Thus, regulation of the proportions of di- vs. tetraether lipids likely constitutes part of the homeoviscous response of Archaea to variations in temperature (Oger and Cario, 2013). We report here that *T. barophilu*s is also capable of regulating the relative proportion of di- and tetraethers as a function of hydrostatic pressure. An increase from 54 to 64% of the relative proportion of archaeol in response to an increase of pressure from atmospheric to 50 MPa has been previously observed in the piezophilic methanogen *Methanocaldococcus jannaschii* (Kaneshiro and Clark, 1995), which is the only example to date of homeoviscous adaptation of the archaeal membrane in response to variations of hydrostatic pressure. Since increasing hydrostatic pressure induces increased lipid compaction, the observed compositional variations can be interpreted in the framework of homeoviscous adaptation of the membrane to varying hydrostatic pressure. Interestingly, in addition to this modification of the ether lipid composition, our results show that the unsaturation level of the apolar lipids of *T. barophilus* increases with increasing pressure and decreasing temperature (**Figure 4**). The regulation of the unsaturation level of membrane lipids is a homeoviscous adaptation strategy frequently observed in psychrophilic bacteria (Wirsen et al., 1986; Yayanos, 1986). Such a strategy has been reported for the psychrophilic archaeon *Methanococcoides burtonii* which accumulates unsaturated archaeol derivatives in response to a lowering in temperature (Nichols et al., 2004). As far as we know, the present work is the first report of the regulation of the unsaturation level of apolar lipids in a thermophile. A similar modification of the degree of unsaturation of apolar lipids has been observed for polyunsaturated squalane derivatives produced by methanogens and halophiles as a function of growth stage and aeration (Langworthy et al., 1972, 1974; Tornabene, 1978). The regulation of the unsaturation level of polyunsaturated lycopane derivatives in *T. barophilus* cells as a function of pressure and temperature conditions could indicate a possible role in homeoviscous adaptation, in addition to the regulation of the ratio of di- vs. tetraether core lipids.

### Are Apolar Lipids of *T. barophilus* Membrane Lipids?

**FIGURE 5:**
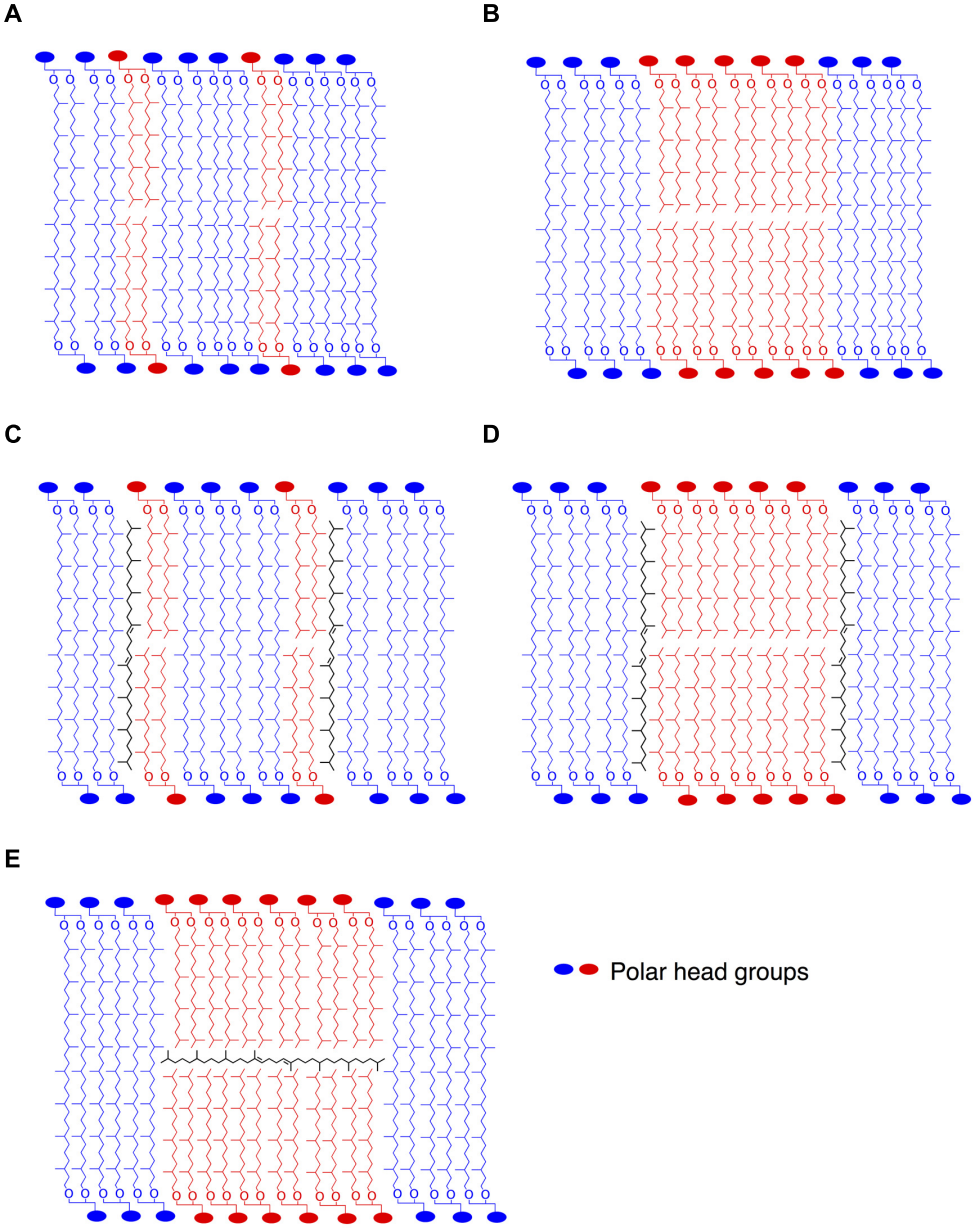
**Stylized schematic representation of lipid organization in putative archaeal membrane models**. Homogenous lipid mix of di- and tetraether lipids **(A)**, coexistence of monolayer and bilayer domains **(B)**. As proposed by Lanyi et al. (1974) or Gilmore et al. (2013), lycopane-derivatives in the hydrophobic region of the membrane in parallel to the isoprenoid chains in a homogenous **(C)** or bilayer domain-containing **(D)** membrane. In contrast, lycopane-derivatives could be inserted in the hydrophobic midplane of bilayer microdomains in parallel to the surface of the membrane **(E)**, as also suggested by Haines (2001) for squalane in membranes of halophiles.

The cellular function of apolar lipids in Archaea, Eukarya, and Bacteria remains unclear. Cellular lipids can fulfill three general functions. First, they are used to form the matrix of cellular membranes. Second, because of their reduced state, they can serve as efficient storage of caloric reserves and stocks of fatty acid components that are needed for membrane biogenesis. Third, lipids can act as first and second messengers in signal transduction and molecular recognition processes (Fernandis and Wenk, 2007; van Meer et al., 2008). The latter function can be ruled out, since there is no evidence of lipid signaling in Bacteria or Archaea. Furthermore, we show that apolar lipids are accumulated in very low concentrations in *T. barophilus* cells, ca. 1–2% of total lipids, which does not support a major role for these lipids as energy storage. It is also unlikely that apolar isoprenoid hydrocarbons of *T. barophilus* could serve as building blocks for archaeal polar lipid biosynthesis or could be derived from polar lipid degradation, since lycopane-type isoprenoid chains are derived from a tail-to-tail condensation of two phytanyl isoprenoid chains, as opposed to the head-to-head condensation of the biphytane isoprenoid chains observed in GDGTs from archaea. The regulation of the level of unsaturation of the lycopane derivatives as a function of pressure and temperature in *T. barophilus* may support a role of these apolar lipids in homeoviscous adaptation and, thus, their presence in membranes. In addition to this species and *Thermococcus litoralis* (Lattuati et al., 1998), irregular polyunsaturated isoprenoid lipids have been observed in methanogens, halophiles and alkaliphiles, but have never been detected in thermoacidophiles (Langworthy, 1982). Experiments performed on archaeal lipids in the presence or absence of apolar lipids revealed that the domain structure in monolayered membranes is strongly influenced by the apolar components (Gilmore et al., 2013). These experiments demonstrated that in these archaeal membrane analogs, squalene plays a function similar to that of cholesterol in eukaryotic membranes, helping to orient the lipid tails away from the interface. This allows the lipid tails to pack more closely together, lowering the average area per molecule. The enhanced order leads to the formation of domains in the membrane in the presence of squalene (Gilmore et al., 2013), and to an increased impermeability of the membrane (Lanyi et al., 1974). In the absence of a midplane interface, squalane derivatives could insert within the hydrophobic domain in parallel to the isoprenoid chains. The location of squalene in bilayered membranes remains a subject of debate. Indeed, Haines (2001) and Hauß et al. (2002) have demonstrated that squalane molecules could sit at the midplane of the lipid bilayers, in parallel to the surface of the membrane. By crowding the inner space of the bilayer, the presence of isoprenoid hydrocarbons in the midplane would explain two specific properties of the halophilic membrane: first the decreased proton and water permeability and second the increased membrane rigidity (Haines, 2001). This proposed ultrastructure is yet to be confirmed in membranes of extreme halophiles. In *T. barophilus*, membrane lipids consist of a majority of tetraether and diether lipids and ca. 1–2% of apolar lipids, which raises questions about the spatial arrangements of these molecules in the membrane. The membrane could be composed of a monolayer matrix constituted by a homogenous mix of tetraether and diether lipids (**Figure 5A**). Alternatively, the membrane could be constituted of coexisting domains of bilayers and monolayers (**Figure 5B**). Polyunsaturated lycopane-derivatives could insert similarly to cholesterol in eukaryotic membranes as proposed by Lanyi et al. (1974) and Gilmore et al. (2013; **Figures 5C,D**). Alternatively, these apolar lipids may insert in the midplane of the bilayer domains formed by diether lipids, parallel to the plane of the membrane as proposed for extreme halophiles by Haines (2001) and Hauß et al. (2002; **Figure 5E**). The presence of a significant proportion of archaeol in *T. barophilus* makes the existence of such bilayer membrane domains plausible. Whether models C, D, or E are valid remains to be addressed. As mentioned above, membrane structures based on models C or D would yield increased membrane rigidity and impermeability under high temperature. In contrast, benefits associated with model E would combine the increased membrane fluidity of the archaeol-based membrane and the increased rigidity and reduced permeability brought by the unsaturated lycopane derivatives present in the midplane of the bilayer. Thus, altering the degree of unsaturation of lycopane derivatives and promoting a bilayer-type membrane could strongly modify the melting transition temperatures and lead to more fluid membrane domains under low temperature or high pressure conditions (Smeller, 2002), and represent a possible adaptation strategy in *T. barophilus*.

## Conclusion

The current study sheds light on homeoviscous adaptation of the membrane lipid composition of one of the major groups of archaeal hyperthermophiles from deep-sea hydrothermal vents. In contrast to previous reports, we show that the tetraether lipid GDGT-0 is among the major core lipids in the piezophilic hyperthermophile *T. barophilus*. We demonstrate that membrane adaptation in hyperthermophilic HHP-adapted Archaea involves the same mechanistic response (e.g., regulation of the relative proportions of di- vs. tetraether lipids) as that previously observed for other environmental parameters such as pH and temperature. We further demonstrate that the structures of non-polar isoprenoid lipids synthesized by this archaeon are also affected by temperature or hydrostatic pressure variations, raising numerous questions regarding the role of these apolar lipids in the structure and adaptation of the membrane of Archaea composed of di- and tetraether lipids. Apolar lipids drastically modify the physical properties of archaeal membrane analogs, but the way they interact with polar lipids remains to be investigated. Amongst possible membrane models that can be proposed for Archaea synthesizing both di- and tetraether lipids, some suggest the existence of micro domains within the membrane into which apolar lipids could be inserted, leading to structures similar to the lipid rafts of eukaryotes. Since membrane properties differ drastically in lipid rafts, the latter are essential for several specific cellular functions. The existence of lipid rafts could help explaining how Archaea with very close membrane lipid compositions cope with very different external environmental conditions. Further studies are required to confirm or infirm the existence of lipid rafts in archaeal membranes.

## Conflict of Interest Statement

The authors declare that the research was conducted in the absence of any commercial or financial relationships that could be construed as a potential conflict of interest.
