# Supplementary material for: Membrane homeoviscous adaptation in the piezo-hyperthermophilic archaeon Thermococcus barophilus
Source: Front Microbiol. 2015 Oct 21;6:1152. doi: 10.3389/fmicb.2015.01152 (PMC4612709; doi:10.3389/fmicb.2015.01152)
Supplement: Supplementary file 1 [file Presentation_1.PDF]

Supplementary Material 1 1

Membrane homeoviscous adaptation in the piezo-hyperthermophilic archaeon Thermococcus barophilus

Anaïs Cario[1], Vincent Grossi[1], Philippe Schaeffer[2], Philippe M. Oger[1*]

[1] Laboratoire de Géologie de Lyon, UMR 5276 CNRS, Ecole Normale Supérieure de Lyon, Université Claude Bernard Lyon 1, 69364 Lyon (France)
[2] Laboratoire de Biogéochimie Moléculaire, CNRS UMR 7177, Institut de Chimie de Strasbourg, Ecole de Chimie, Polymères et Matériaux, Université de Strasbourg, 67200 Strasbourg (France)

*** Correspondence:** Philippe M. Oger, Laboratoire de Géologie de Lyon, UMR CNRS 5276, Ecole Normale Supérieure de Lyon, 46, Allée d'Italie, F-69342, France.
poger@ens-lyon.fr (P.M. Oger)

Keywords: Archaeal lipids, archaeal membrane, homeoviscous adaptation, piezophily, deep-biosphere, GDGT-0, *Thermococcus barophilus*.

First Author*, Co-Author, Co-Author

*** Correspondence:** Corresponding Author: email@uni.edu

1 Supplementary Figures

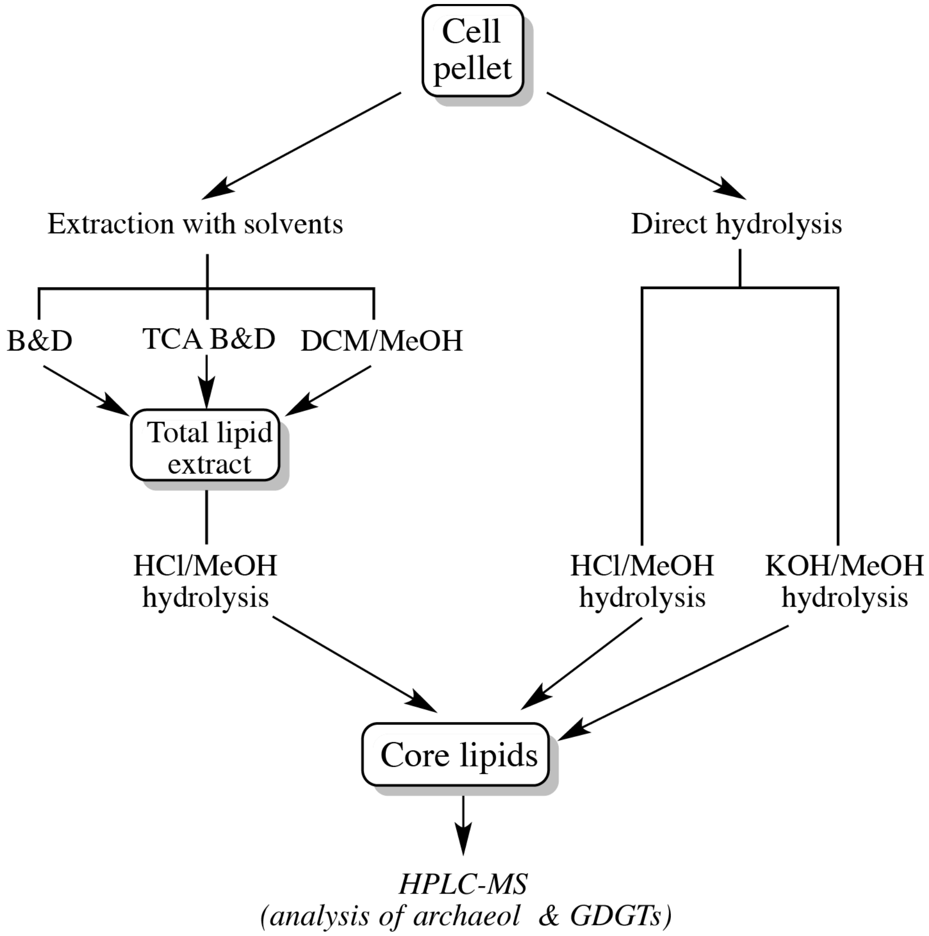

Supplementary Figure 1. : Experimental scheme showing the different analytical procedures tested for the extraction of core lipids from *T. barophilus*. B&D, Bligh and Dyer; TCA, trichloroacetic acid acidified B&D; DCM/MeOH; dichloromethane:methanol, 1:1, v/v; HCl/MeOH, acid hydrolysis; KOH/MeOH, base hydrolysis.

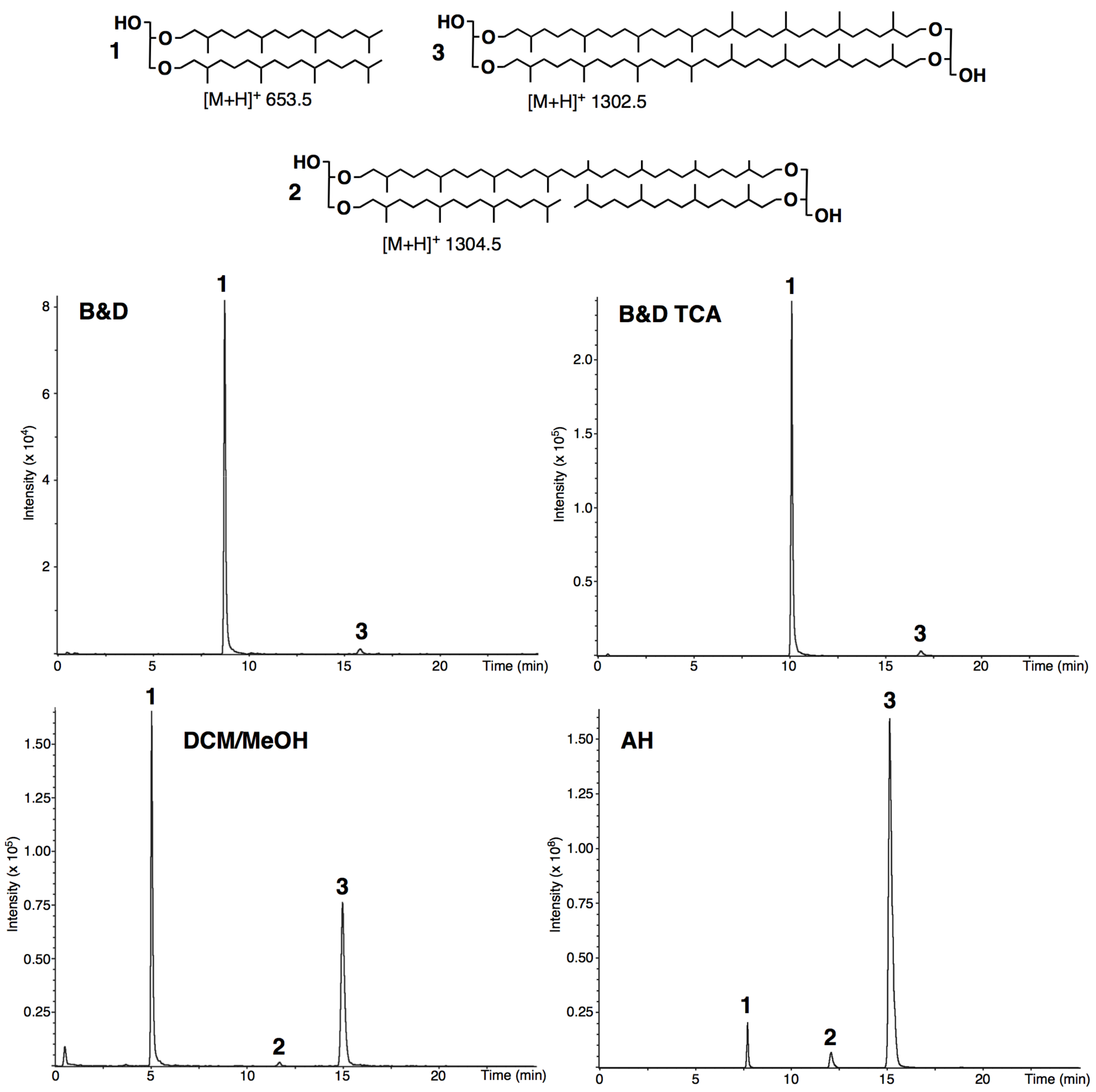

Supplementary Figure 2. : HPLC-MS chromatograms of *T. barophilus* core lipids, following different extraction procedures. 1: Diphytanyl glycerol diether, DPG (archaeol); 2: Glycerol trialkyl glycerol tetraether, GTGT; 3: Glycerol dibiphytanyl glycerol tetraether, GDG T-0; B&D, Bligh and Dyer; TCA, trichloroacetic acid acidified B&D; DCM/MeOH; dichloromethane:methanol, 1:1, v/v; AH, acid hydrolysis.
